# Anthropometric indices and cut-off points for screening of metabolic syndrome among South African taxi drivers

**DOI:** 10.3389/fnut.2022.974749

**Published:** 2022-08-11

**Authors:** Machoene Derrick Sekgala, Maretha Opperman, Buhle Mpahleni, Zandile June-Rose Mchiza

**Affiliations:** ^1^School of Public Health, University of the Western Cape, Bellville, South Africa; ^2^Human and Social Capabilities, Human Sciences Research Council, Cape Town, South Africa; ^3^Functional Food Research Unit, Department of Biotechnology and Consumer Science, Cape Peninsula University of Technology, Cape Town, South Africa; ^4^Non-Communicable Diseases Research Unit, South African Medical Research Council, Cape Town, South Africa

**Keywords:** metabolic syndrome, anthropometric indices, a body shape index (ABSI), body roundness index (BRI), waist circumference, body mass index (BMI), waist-to-height ratio (WHtR), receiver operating characteristic curve

## Abstract

**Background:**

Detecting the early onset of metabolic syndrome (MetS) allows for quick intervention which may slow progression to a variety of health consequences, hence, determining the best measurement to detect MetS is essential.

**Aim:**

This research aimed at examining the MetS predictive power of anthropometric indices, such as body mass index (BMI), waist circumference (WC), waist-to-height ratio (WHtR), body shape index (ABSI), body roundness index (BRI), percentage body fat (%BF), conicity index (CI), and Clínica Universidad de Navarra-body adiposity estimator (CUN-BAE) to determine the cut-off points to identify male South African taxi drivers with MetS.

**Method:**

A cross-sectional study was conducted among 185 male taxi drivers. Their weight, height, WC, blood lipid profile were measured. International Diabetes Federation (IDF) definition was used to define MetS. Receiver Operating Characteristic (ROC) curves were used to compare the predictive ability of Anthropometric indices to detect MetS.

**Results:**

The mean age of the participants was 39.84 years. Overall, 41.6% (N = 77) of the participants presented with MetS. The mean values for BMI, WC, WHtR, %BF, BRI, CUN-BAE, ABSI and CI were 28.60 ± 6.20 kg/m^2^, 99.13 ± 17.59 cm, 0.58 ± 0.10, 27.28 ± 8.28%, 5.09 ± 2.33, 27.78 ± 8.34, 0.08 ± 0.01 and 1.70 ± 0.19, respectively. The mean values for these indices were significantly (*p* < 0.001) higher in participants with MetS. The highest area under the curve (AUC) outcomes for screening MetS were for the %BF and CUN-BAE, followed by the BMI and WHtR, and lastly the BRI. All these anthropometric indices had outstanding discriminatory powers for predicting MetS with AUCs and sensitivity values above 80%. The BMI, WHtR, %BF, BRI, and CUN-BAE, had cut-off points for detection of metS in South African men at 28.25 kg/m^2^, 0.55, 25.29%, 4.55, and 27.10, respectively. Based on the logistic regression models abnormal BMI, WHtR, %BF, BRI, CUN-BAE, TG, FBG, systolic BP, diastolic BP and WC showed increased risk of MetS.

**Conclusion:**

While the %BF, CUN-BAE, BMI, WC, WHtR, BRI, CI and CUN-BAE could predict MetS among South African male taxi drivers, these indices were less effective in predicting the individual MetS risk factors such as TG, BP, and FBG.

## Introduction

Metabolic syndrome (MetS) is a cluster of multiple, interconnected metabolic risk factors that promote the development of non-communication diseases (NCDs) such as diabetes, abdominal obesity, high cholesterol, low high-density lipoprotein cholesterol (HDL-c), and high blood pressure (1). Several international studies report an increased prevalence of MetS among occupational drivers when compared to other professionals such as industrial and office workers (2–4). International evidence further suggests that occupational drivers are at increased risk of cardiovascular diseases (5, 6). In South Africa there is dearth of data on the prevalence of MetS among occupational drivers and more specifically minibus taxi drivers (hereafter referred to as taxi drivers). Ramukumba and Mathikhi (7) state that taxi drivers' working environment is characterized by poor eating habits, elevated stress levels caused by long hours of driving, exposure to various environmental hazards such as air pollution as well as a lack of exercise. Their poor eating habits are aggravated by regular consumption of fried foods and snacks high in sugar and salt since these foods are relatively cheap and easily accessible at taxi ranks and bus stations where they operate (8, 9). Additionally, a recent study in Cape Town reported a notable prevalence of central obesity among taxi drivers as they overconsume alcohol and smoke to overcome stress (10).

Metabolic syndrome is regarded as a public health issue that is associated with the clustering of a wide variety of risk factors that co-exist in an individual (11, 12). The World Health Organization (13), the European Group for the Study of Insulin Resistance (EGIR) (14), the National Cholesterol Education Program Adult Treatment Panel III (15), the American Association of Clinical Endocrinology (AACE) (16, 17), International Diabetes Federation (IDF) (18) and American Heart Association/National Heart, Lung, and Blood Institute (AHA/NHLBI) (19) use different algorithms to determine MetS. These algorithms are based on the risk factors considered to be clinically realistic assessment measures for MetS for the specific populations that are under study (20). In general, any MetS algorithm includes a combination of three or more risk factors, namely: body mass index (BMI), central obesity, insulin resistance, glucose intolerance and diabetes, elevated triglycerides (TG), low levels of HDL-c, and hypertension.

In rural South Africa, the MetS prevalence in men ranges from 7.9 to 17.9% of which 7.9 and 10.5% was reported by Motala et al. (21) and Motala et al. (22), respectively among individuals aged >15 years, in a rural African (black) community of Zulu descent in the Ubombo district of the province of KwaZulu-Natal. Peer et al. (23) on the other hand reported a 17.9% prevalence among black men living in Cape Town, while Sekgala et al. (24) reported it to be 8.6% in young black South African men aged 18–30 years living in Limpopo, a rural province of South Africa.

Obesity and overweight are two important risk factors for MetS (25). According to Suliga et al. (26) and Kabała and Wilczyński (27), the BMI is the most common metric for determining obesity as it is simple to calculate and has well-defined cut-off points. This index is utilized in research all around the world as it is non-invasive. This makes it the best index that allows for possible comparisons of nutritional statuses in different populations globally. However, its inability to portray sex dimorphism, including ethnic differences in adiposity, adipose tissue distribution, and age-related body composition limits the BMI for measuring MetS in different populations (28). Hence, researchers prefer to use anthropometrical measures that show adipose tissue distribution, differentiate central or abdominal obesity when classifying MetS, WC and percentage body fat (%BF), to be specific.

There is limited data on the central obesity status of South African men in the taxi driving industry. This is despite the substantiated international evidence (4, 29) suggesting that 50% of male occupational drivers display significantly higher depositions of visceral adipose tissue compared to the general male population. Visceral adipose tissue (also known as central/abdominal obesity) is a hormonally active component of total body fat, which possesses unique biochemical characteristics that influence several pathological processes in the human body (30) including the development of non-communicable diseases (NCDs) (31).

The deposition of visceral adipose tissue is measured using both invasive (32) and non-invasive anthropometrical measurements (33). Among the most common, non-invasive, and acceptable anthropometrical measurements undertaken to measure central/abdominal obesity and adipose tissue composition are the WC (34) and the %BF (35). Aside from using solely the WC to determine abdominal obesity, researchers often apply WC in the algorithms to measure its relationship with height (WHtR) (34, 36), as well as the BMI to measure the conicity index (CI) (37), body roundness index (BRI) (36) and a body shape index (ABSI) (38). International studies further suggest that the results of central obesity assessments measured by WC show the strongest connections with metabolic risk variables (39, 40).

There are four skin fold measurements (biceps, triceps, subscapular and suprailiac) that are commonly used in clinical interventions to measure %BF which, according to Rodriguez-Escudero et al. (35) is a good indicator for body composition. Aside from using solely the skinfold measurements to measure %BF, the Clínica Universidad de Navarra-Body Adiposity Estimator (CUN-BAE) is a measure that applies the outcome of %BF to an algorithm that compares it to the individual's BMI (28). The CUN-BAE is based on the BMI, but it has the added benefit of accounting for age and gender body composition differences, hence it is regarded as the best index for determining the %BF. The CUN-BAE has also been significantly associated with the actual adipose tissue composition (28) and is therefore a useful tool for identifying the risk of MetS.

Even though multiple articles on the link between adiposity and the risk of MetS have been published, it is still difficult to determine unambiguously the best measure to be applied in an algorithm to identify individuals with MetS, especially in South Africa. To our knowledge, there has never been a study conducted in South Africa to assess the ability of different anthropometric indices to detect MetS, as well as determine their cut-off point to screen for MetS among male taxi drivers. Hence, the current study was long overdue. This study aimed to examine the MetS predictive power of anthropometric indices such as the BMI, WC, WHtR, ABSI, BRI, %BF, CI, and CUN-BAE, and determine the cut-off points to identify male South African taxi drivers at risk of MetS. The outcomes of this research will inform policies directed at improving the health status of South African taxi drivers.

## Materials and methods

### Study design

This cross-sectional study was conducted among 185 conveniently sampled commercial taxi drivers aged 20 years and older who were recruited from the Bellville and Cape Town taxi ranks. These taxi ranks were chosen because they are the two busiest transport interchange areas in the Cape metropole area in the Western Cape Province of South Africa (8).

### Study participants and sample size

All taxi drivers who were available, willing to participate and those who met the inclusion criteria were included in the study. Eligible participants had to be 20 years and older, fluent in English and/or Afrikaans and/or IsiXhosa (the most spoken languages in Cape Town and surrounding areas), able to provide informed consent, and willing to donate a blood sample for metabolic assessments. Taxi drivers who have at least 1-year experience as a driver around the targeted interchange areas in the Western Cape Province and also being a members of a recognized taxi association. Only men were included in the study given that more than 99% of taxi drivers operating in these transport interchange areas were men. Participants who were on any form of chronic medication and/or with chronic diseases history were excluded.

Since no similar studies on MetS prevalence among taxi drivers in Cape Town could be located and the fact that the proposed study focused on taxi drivers (>80% black men), the sample size was based on the findings of Peer et al. (23) who indicated a 17.9% MetS prevalence among black men in Cape Town. As such, the sample size was obtained using the formula by Daniel and Cross (41) for cross-sectional studies.


$$\begin{array}{l} \text{Sample Size }\left(\text{N}\right):\;{\text{Z}}^{2*}\;{\left(\text{p}\right)}^{*}\;\left(1-\text{p}\right)/{\text{c}}^{2} \end{array}$$

Where: Z = Z value (e.g., 1.96 for 95% confidence level); p = expected proportion of the population, expressed as decimal=0.179; c = confidence interval, expressed as decimal = 0.05.

Therefore, the estimated sample size was N = 226 and after adjusting for 5% non-response the sample size increased to N = 237.

**Figure 1:**
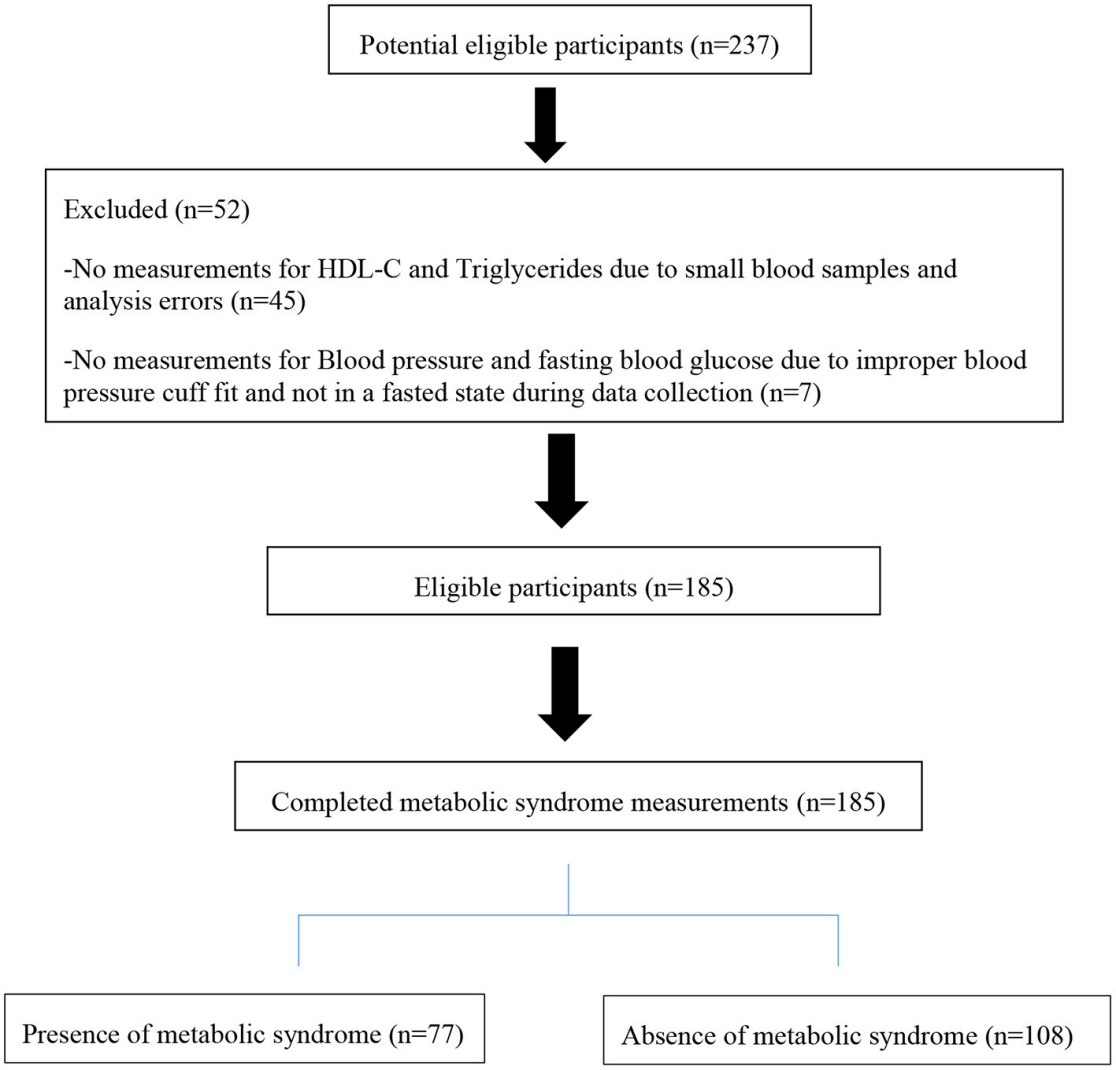
Flow diagram of participants' selection for this study.

Of the 237 participants who agreed to participate, only 185 agreed to complete all the measurements and donate blood specimens for the metabolic parameters (see Figure 1).

### Socio-demographic data

Socio-demographic data and information on the participants' lifestyles (duration of sleep, physical activity, alcohol consumption and cigarette smoking) were collected *via* face-to-face interviews using a structured and previously validated questionnaire (30). Collected socio-demographic variables included age, socio-economic status (defined based on the household income, marital status, and education level).

### Measurements

#### Anthropometric indicators

Weight, height and WC were measured to calculate the anthropometric indices using standard procedures (42). All measurements were conducted by a qualified dietitian, with the help of qualified and trained field nurses.

Skinfold thickness was measured on both sides of the body using a Lange Skinfold Caliper at four locations: biceps, triceps, subscapular, and suprailiac. The biceps skinfold thickness was measured at the midpoint of the arm while the individual sat in a supine position with arms relaxed and resting on the thighs. Triceps skinfold thickness was measured in the sitting position with arms crossed at a 90° bend and resting on thighs at the midpoint between the acromion and the olecranon process. The subscapular skinfold was measured while standing with arms to the side. The shoulder blade was located and followed down to the point where it began to curve. The skin was pinched and the calipers were used to measure the skinfold. Still in the standing position the suprailiac skinfold was also measured. The skin above the right hipbone was measured along the midaxillary line (43).

Waist circumference was measured in cm above the iliac crest and below the lowest rib margin at minimum respiration by the use of a non-stretch tape measure (44). Height was measured in meters to the nearest cm using a SECA stadiometer with a right-angle headboard wide enough to rest across the top of the head. The participants were measured without shoes and standing up-right, feet together, knees straight, and heels, buttocks, and shoulder blades in contact with the stadiometer (45). Weight was measured to the closest hundredth of a gram using an electronic scale that was calibrated before use with a total calibration weight of 200 kg. Weight was measured while the participants were standing in the center of the scale and looking straight ahead with minimal clothing (46).

Based on the afore-mentioned measurements, the following indicators were calculated:

a. BMI = weight (kg) / height^2^ (m^2^): <18 kg/m^2^, 18–24.9 kg/m^2^, 25–29.9 kg/m^2^, and ≥ 30 kg/m^2^ considered as underweight, normal weight, overweight and obese, respectively (38).
b. WHtR = WC (cm)/height (cm), The WHtR of > 0.5 was considered abnormal (47).
c. ABSI = WC (m)/[BMI^2/3^(kg/m^2^) ^*^ height^1/2^ (m)] (38). The ABSI of >0.07 was considered abnormal.
d. CI = 0.109^−1^ WC (m) [weight (kg)/height (m)]^−1/2^. The CI of >1.25 was considered abnormal (37).
e. $BRI=364.2\;-\;365.5\;x\;\sqrt{\left(1-\frac{\left(\frac{WC}{2\pi }\right)2}{\left(0.5Xheight\;\right)2}\right)}$
  BRI of >3.5 was considered abnormal (35).
f. %BF= (495/Body Density) - 450 (35). The %BF of >25.00 is considered abnormal (48). %BF was calculated based on the average skinfold thickness measurement from each of the four sites.
g. CUN-BAE was calculated using the equation %BF = - 44.988 + (0.503 x age) + (10.689 x sex) + (3.172 x BMI) - (0.026 x BMI^2^) + (0.181 x BMI x sex) - (0.02 x BMI x age) - (0.005 x BMI^2^ x sex) + (0.00021 x BMI^2^ x age), where age is measured in years, and sex was codified as 0 for men. A CUN-BAE of >20.00 is considered abnormal (28).

#### Blood pressure and blood biochemical parameters

Blood pressure was measured using an Omron BP monitor (Model M3 Intellisense, Mannheim, Germany). Blood pressure was measured on the artery of the right upper limb when the individual was seated and rested at ground level. Following a 5-min rest period in a sitting position BP was measured twice with at least a 5-min interval apart. The average of the 2 measurements was considered for data analysis. Hypertension was defined as systolic blood pressure (SBP) > 130 mm Hg or diastolic blood pressure (DBP) > 85 mmHg (49).

#### Metabolic parameters

Blood was sampled from participants by qualified field nurses in the morning after a 12-h overnight fast and was kept on dry ice and transported to the laboratory for processing. On arrival at the lab, the blood specimens were centrifuged for 5 min at 2,500 rpm at room temperature to separate the plasma and red blood cells. The concentration of TGs was assessed using the phosphoglycerides oxidase peroxidase method while HDC-C was obtained using the colorimetric non-precipitation method. Plasma was used for analysis. The glucose concentration was estimated by the capillary method using a glucometer (One Touch^®^).

#### Definition of metabolic syndrome

Following the criteria established by the International Diabetes Federation (IDF) Task Force on Epidemiology and Prevention (joint interim statement in 2009) (49), MetS was defined as the presence of three or more of the following five NCDs: abdominal obesity (WC > 94 cm) in males; FBG ≥ 5.5 mmol/L; TGs ≥1.7 mmol/L; HDL-c <1.0 mmol/L in males and SBP ≥130 mmHg or DBP ≥85 mmHg.

### Ethical approval

This study was approved by the Biomedical Science Research Ethics Committee of the University of the Western Cape (Reference number: BM18/9/25), the City of Cape Town (CCT), and the Western Cape Department of Health. Permission to collect data from the participants was granted by the taxi rank coordinators affiliated with the Western Cape Taxi Drivers' associations. Taxi drivers were informed about the details of the study, what would be expected of them, and that they could withdraw from the study at any time and no punitive measures will be taken against them if they chose to do so. Those who were willing to participate were provided information sheets with details of the research and the contacts they could use in case of further information or to lodge disputes. They were then invited to provided written consent before the commencement of this study. Their rights for data confidentiality and anonymity were ensured throughout the study.

### Statistical analysis

All data were analyzed using the Statistical Package for Social Science (IBM-SPSS, version 24.0 for Windows; SPSS Inc., Chicago, IL, USA). All continuous variables were expressed as means and standard deviations (Mean±SD) while categorical variables were reported as frequencies and percentages (N and %). To measure the relationship between dependent and independent variables the *t-test* was used for continuous variables and the chi-square test for categor-ical variables.

The Receiver Operating Characteristic (ROC) curve analyses were used to compare the MetS predictive abilities of different anthropometric indices and to determine the optimal cut-off values. Using the same method, the area under the curve (AUC) with 95% Confidence Intervals (CIs) were also estimated. The AUC was used to measure the accuracy for each anthropometric index to discriminate between individuals who presented with MetS and those who did not. The AUC values between ≥0.5 and <0.6 (50 and 60%), ≥0.6 and <0.7 (60 and 70%), ≥0.7 and <0.8 (70–80%), and ≥0.8 and ≥0.9 (80–90%) were regarded to have poor, acceptable, excellent and outstanding abilities to predict MetS, respectively (50). The best cut-off points were determined as those closest to the upper left angle of the ROC curve (51). In this approach, the lowest cut-off value corresponds to a Sensitivity = 1 and Specificity = 0. Until a cut-off value corresponding to a test Sensitivity = 0 and Specificity = 1 is reached, the test Sensitivity declines, and the test Specificity increases as the cut-off value rises. There is a cutoff value over this interval at which the test's sensitivity and specificity are equal. As a result, the criterion for determining the test cut-off value that corresponds to this specific point where Sensitivity = Specificity is the one that is used. This point is analytically the intersection of the line connecting the left-upper corner and the right-lower corner of the unit square (the line Sensitivity = Specificity) of the ROC curve. Logistic regression analysis was applied to calculate the association between each of the anthropometric indices (BMI, WC, WHtR, %BF, BRI, CUN-BAE, ABSI and CI), MetS and its risk factors. Combinations of several indices were investigated to comprehensively predict the risk of MetS among taxi drivers The associations were presented as odds ratios (ORs) with CI that did not overlap and *p* < 0.05 showing significant differences between the OR outcomes. The OR outcomes were also adjusted by age group, race, employment, province, locality, education, smoking, alcohol intake and physical activity. Three logistic regression models were applied: *model 1*, adjusted for age; *model 2*, adjusted for age, race, marital status, driving experience in years, and education; and *model 3*, further adjusted for age, race, marital status, driving experience in years, education, smoking, alcohol intake and physical activity. *P* < 0.05 and CIs that did not overlap were assumed statistically significant for all other calculations.

## Result

Table 1 presents the sociodemographic and anthropometric characteristics of 185 male participants who completed the study.

**Table 1:** Sociodemographic and anthropometric characteristics by the presence/absence of MetS among the taxi drivers in Western Cape, South Africa.

	**Total** ***N* = 185**	**MetS** **present *n* = 77**	**MetS** **absent *n* = 108**	
	**mean ±SD**	**mean ±SD**	**mean ±SD**	***P*** **-value**
Age (years)	39.84 ± 10.46	43.73 ± 10.34	37.27 ± 10.21	<0.001
Race *n* (%)				0.932
Black Non-black	146 (78.9) 39 (21.1)	33 (42.9) 44 (57.1)	85 (78.7) 23 (21.3)	
Merital status *n* (%)				0.279
Single, divorced, separated or widowed Married, or living as married	88 (47.3) 97 (52.7)	33 (42.9) 44 (57.1)	55 (50.9) 53 (49.1)	
Driving experience (years) *n* (%)				<0.001
1–7 8>	103 (55.7) 82 (44.3)	31 (40.3) 46 (59.7)	72 (66.7) 36 (33.3)	
Educational level *n* (%)				0.714
No schooling or primary Some high school and higher education	58 (31.4) 127 (68.6)	23 (29.9) 54 (70.1)	35 (32.4) 73 (67.6)	
BMI (kg/m^2^)	28.60 ± 6.20	32.71 ± 5.88	25.65 ± 5.21	<0.001
WC (cm)	99.13 ± 17.59	110.83 ± 16.72	90.72 ± 14.50	<0.001
WHtR	0.58 ± 0.10	0.64 ± 0.09	0.53 ± 0.09	<0.001
Weight (kg)	84.74 ± 19.67	97.35 ± 18.66	75.52 ± 16.41	<0.001
Height (cm)	172.03 ± 7.93	172.59 ± 9.33	171.44 ± 7.23	0.387
%BF	27.28 ± 8.28	33.11 ± 7.63	23.16 ± 6.85	<0.001
ABSI	0.0812 ± 0.0840	0.0829 ± 0.0901	0.0800 ± 0.00775	<0.001
BRI	5.09 ± 2.33	6.68 ± 2.50	4.06 ± 1.81	<0.001
CUN-BAE	27.78 ± 8.34	33.55 ± 6.61	23.53 ± 7.52	<0.001
CI	1.70 ± 0.19	1.78 ± 0.21	1.65 ± 0.17	<0.001
Biceps	10.66 ± 6.73	12.84 ± 6.06	8.53 ± 3.31	<0.001
Triceps	17.41 ± 8.75	20.56 ± 9.53	14.95 ± 7.40	<0.001
Subscapular	26.16 ± 13.58	32.14 ± 13.76	21.21 ± 10.87	<0.001
Suprailiac	24.20 ± 13.08	29.40 ± 11.07	19.40 ± 10.90	<0.001

*BMI, Body Mass index; WC, Waist circumference; WHtR, waist-to-height Ratio; %B, percentage body fat; ABSI, a body shape index; BRI, body roundness index; CUN-BAE, Clínica Universidad de Navarra-Body Adiposity Estimator; CI, Conicity index; MetS, metabolic syndrome. The numerical values are presented as mean ± standard deviation and intergroup compared using the Mann-Whitney U test*.

The Mean ± SD age of the participants was 39.84 ± 10.45 years. The mean values for BMI, WC, WHtR, %BF, BRI, CUN-BAE, ABSI and CI were 28.60 ± 6.20 kg/m^2^, 99.13 ± 17.59 cm, 0.58 ± 0.10, 27.28 ± 8.28%, 5.09 ± 2.33, 27.78 ± 8.34, 0.08 ± 0.01 and 1.70 ± 0.19, respectively. Overall, 41.6% participants presented with MetS, while those with MetS were significantly older (*p* < 0.001) than those without MetS (mean age of 43.73 ± 10.34 vs. 37.27 ± 10.21 years).

The mean values for BMI, WC, WHtR, %BF, BRI, CUN-BAE, ABSI and CI were significantly (*p* < 0.001) higher in participants with MetS compared to those without MetS. The mean values for all 4 skinfolds were significantly (p < 0.001) higher among participants with MetS than those without MetS.

Table 2 presents mean NCD risk factors by the presence/absence of metabolic syndrome. Participants who presented with MetS displayed significantly (*p* < 0.001) higher mean values for TG (1.88 ± 1.49 vs. 0.96 ± 0.45), FBG (7.87 ± 4.82 vs. 5.33 ± 1.13), SBP (141.47 ± 18.79 vs. 127.40 ± 13.33) and WC (110.83 ± 16.72 vs. 90.72 ± 14.50) compared to those without MetS. Participants who presented with MetS displayed significantly lower mean values for HDL-c compared to those without MetS (1.00 ± 0.28 vs. 1.20 ± 0.36, *p* < 0.001).

**Table 2:** Mean non-communicable disease risk factors by the presence/absence of MetS.

**Risk factors of MetS**	**Total**	**MetS** **present *n* = 77**	**MetS** **absent *n* = 108**	**Intergroup comparison *p*-value**
	**mean ±SD**	**mean ±SD**	**mean ±SD**	
Triglycerides (mmol/L)	1.35 ± 1.12	1.88 ± 1.49	0.96 ± 0.45	<0.001
HDL-c (mmol/L)	1.11 ± 0.34	1.00 ± 0.28	1.20 ± 0.36	<0.001
FBG (mmol/L)	6.50 ± 3.44	7.87 ± 4.82	5.33 ± 1.13	<0.001
SBP (mmHg)	133.44 ± 17.17	141.47 ± 18.79	127.40 ± 13.33	<0.001
DBP (mmHg)	84.71 ± 13.08	92.73 ± 13.94	79.07 ± 9.12	<0.001
WC (cm)	99.13 ± 17.59	110.83 ± 16.72	90.72 ± 14.50	<0.001

*HDL-c, high-density lipoprotein cholesterol; FBG, fasting blood glucose; SBP, systolic blood pressure; DBP, diastolic blood pressure; WC, waist circumference; MetS, metabolic syndrome. Values are reported as the mean ± standard deviation*.

Table 3 shows the distribution of normal/abnormal proportions of different risk factors for MetS. Abnormal values were recorded for TGs (20.8%), HDL-c (51.6%), FBG (51.7%), SBP (59.7%), DBP (43.3%), BP (35.5%) and WC (59.9%) Based on the participants who had abnormal risk factor outcomes significantly more of them also presented with clustering of these risk factors (presented with MetS) when compared to those who had normal risk factor outcomes [TG (40.3 vs. 6.5%), HDL-c (75.3 vs. 34.3%), FBG (76.6 vs. 30.6%,) SBP (71.4 vs. 49.1%), DBP (70.1 vs. 26.0%), BP (70.1 vs. 14.8%) and WC (92.2 vs. 36.1%)].

**Table 3:** Risk factors of MetS grouped by the presence and absence of MetS.

**Components of MetS**		**Total**	**MetS** **present *n* = 77**	**MetS** **absent *n* = 108**	**Intergroup comparison**
		**N (%)**	**N (%)**	**N (%)**	***p*** **-value**
Triglycerides (mmol/L)	Normal Abnormal	152 (79.2) 40 (20.8)	46 (59.7) 31 (40.3)	101 (93.5) 7 (6.5)	<0.001
HDL-c (mmol/L)	Normal Abnormal	93 (48.4) 99 (51.6)	19 (24.7) 58 (75.3)	71 (65.7) 37 (34.3)	<0.001
FBG (mmol/L)	Normal Abnormal	111 (48.3) 119 (51.7)	18 (23.4) 59 (76.6)	75 (69.4) 33 (30.6)	<0.001
SBP (mmHg)	Normal Abnormal	93 (40.3) 138 (59.7)	22 (28.6) 55 (71.4)	55 (50.9) 53 (49.1)	0.003
DBP (mmHg)	Normal Abnormal	131 (56.7) 100 (43.3)	23 (29.9) 54 (70.1)	82 (75.9) 24.1 (26.0)	<0.001
Hypertension	Normal Abnormal	149 (64.5) 82 (35.5)	28 (29.9) 54 (70.1)	92 (85.2) 16 (14.8)	<0.001
WC (cm)	Normal Abnormal	95 (40.1) 142 (59.9)	6 (7.8) 71 (92.2)	69 (63.9) 39 (36.1)	<0.001

*HDL-c, high-density lipoprotein cholesterol; FBG, fasting blood glucose; SBP, systolic blood pressure; DBP, diastolic blood pressure; WC, waist circumference; MetS, metabolic syndrome. Values are reported as the mean ± standard deviation*.

**Figure 2:**
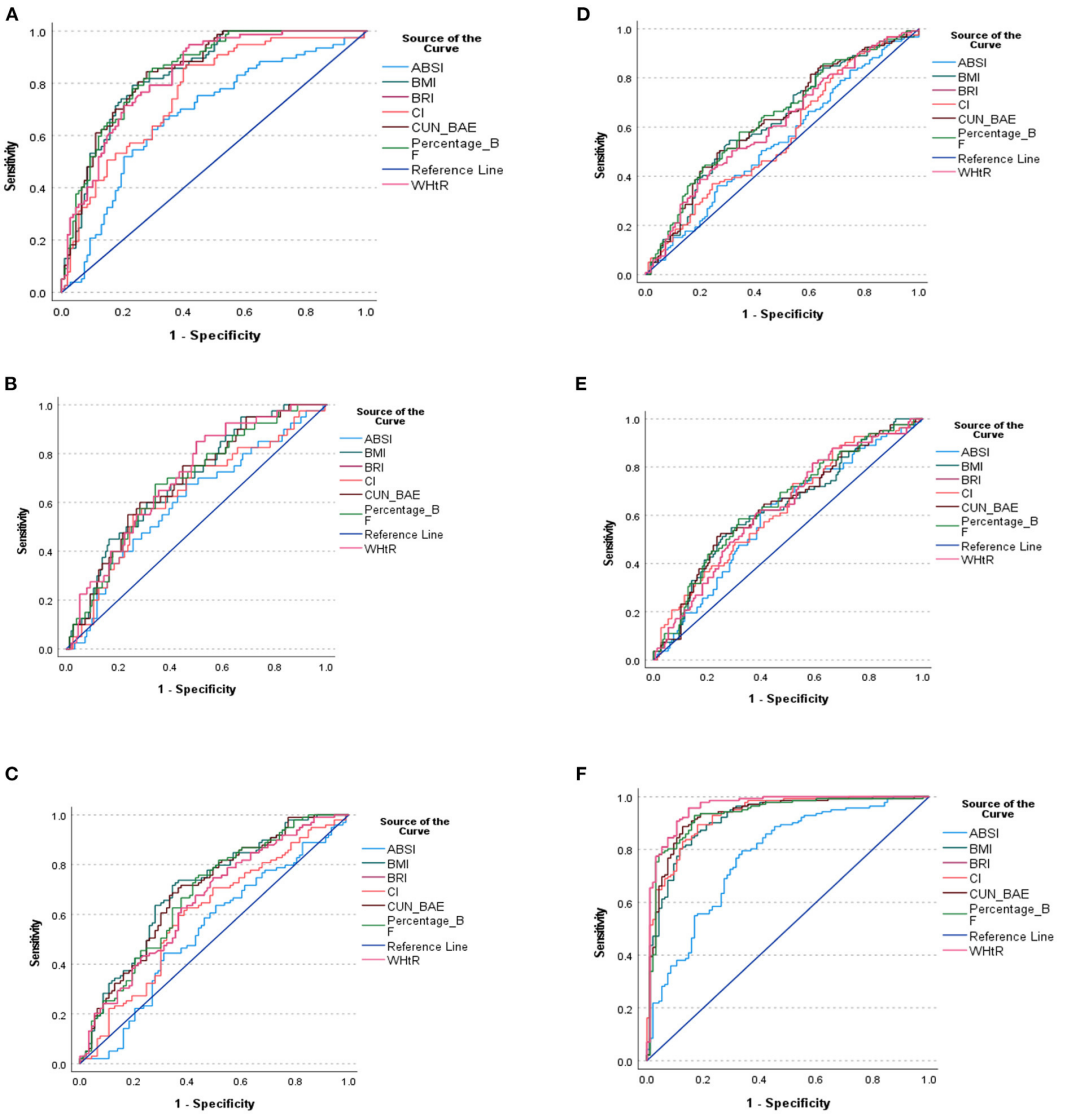
**(A)** ROC curve of the anthropometric indices for the prediction of MetS. **(B)** ROC curve of the anthropometric indices for the prediction of triglyceride. **(C)** ROC curve of the anthropometric indices for the prediction of HDL-C. **(D)** ROC curve of the anthropometric indices for the prediction of FBG. **(E)** ROC curve of the anthropometric indices for the prediction of Hypertension. **(F)** ROC curve of the anthropometric indices for the prediction of WC. **(A–F)** shows the ROC curve of the anthropometric indices cut off points for the prediction of MetS and its components.

Based on Table 4, the most sensitive AUC outcomes for screening MetS were for the %BF (84.8%) and CUN-BAE (84.6%) followed by the BMI (83.8%) and WHtR (83.2%), and lastly the BRI (83.2%). All these indices displayed outstanding discriminatory power for predicting MetS since their AUCs and sensitivity values were all above 80%. The BMI, WHtR, %BF, BRI, and CUN-BAE, cut-off points for detection of MetS in this group were 28.25 kg/m^2^, 0.55, 25.29%, 4.55, and 27.10, respectively. While the CI showed the excellent AUC (76.2%) for predicting the MetS with the cut-off point of 1.70 and the sensitivity of 74% the ABSI only showed acceptable discriminatory power for predicting MetS, with an AUC of 67.7%, and the cut-off point of 0.8, while its sensitivity was 70.1%. The virtualization of the anthropometric indices cut off points for the prediction of MetS and its components is shown in Figures 2A–F.

**Table 4:** Area under the curves (AUC) and cut-off points for the anthropometric indices for the prediction of MetS and its risk factors.

**Anthropometric indices**	**MetS and risk factors**	**AUC**	**95% CI**	***P*** **-value**	**Cut-off point**	**Sensitivity**	**Specificity**
BMI (kg/m^2^)	MetS (IDF criterion)	83.8%	0.782–0.895	<0.001	28.25	80.5%	25.0%
WHtR		83.2%	0.775–0.889	<0.001	0.55	87.0%	36.1%
%BF		84.8%	0.794–0.902	<0.001	25.29	85.7%	29.6%
ABSI		67.7%	0.599–0.756	<0.001	0.08	70.1%	38.9%
BRI		83.2%	0.775–0.889	<0.001	4.55	80.5%	36.1%
CUN-BAE		84.6%	0.791–0.901	<0.001	27.10	84.4%	27.8%
CI		76.2%	0.694–0.831	<0.001	1.70	74.0%	36.1%
BMI (kg/m^2^)	Triglycerides (mmol/L)	67.8%	0.588–0.768	0.001	28.69	63.2%	39.5%
WHtR		69.3%	0.606–0.780	<0.001	0.57	71.1%	44.2%
%BF		60.5%	0.577–0.761	0.001	25.57	71.1%	46.2%
ABSI		69.3%	0.506–0.705	0.046	0.08	60.5%	40.8%
BRI		69.3%	0.606–0.780	<0.001	5.25	60.5%	34.0%
CUN-BAE		67.6%	0.586–0.767	0.001	29.19	60.5%	36.7%
CI		63.4%	0.535–0.732	0.011	1.71	60.5%	38.8%
BMI (kg/m^2^)	HDL-C (mmol/L)	70.9%	0.634–0.784	<0.001	27.74	70.5%	34.4%
WHtR		65.0%	0.582–0.738	<0.001	0.57	60.0%	37.8%
%BF		69.0%	0.614–0.766	<0.001	25.35	67.4%	36.7%
ABSI		53.7%	0.453–0.621	0.384	0.081	50.5%	43.3%
BRI		66.0%	0.582–0.738	<0.001	4.77	60.0%	37.8%
CUN-BAE		70.2%	0.627–0.777	<0.001	26.85	70.5%	35.6%
CI		60.3%	0.521–0.685	0.015	1.71	60.0%	36.7%
BMI (kg/m^2^)	Fasting glucose (mmol/L)	62.5%	0.544–0.706	0.003	27.74	60.9%	46.2%
WHtR		61.3%	0.532–0.694	0.008	0.57	57.6%	44.1%
%BF		64.5%	0.566–0.725	0.001	25.68	60.9%	40.9%
ABSI		55.3%	0.470–0.636	0.214	0.081	52.2%	45.2%
BRI		61.3%	0.532–0.694	0.008	4.88	54.3%	39.8%
CUN-BAE		63.5%	0.555–0.716	0.001	27.25	60.9%	40.9%
CI		57.3%	0.491–0.656	0.085	1.72	42.4%	37.6%
BMI (kg/m^2^)	BP (mmHg)	64.0%	0.558–0.722	0.002	27.44	64.6%	49.2%
WHtR		63.3%	0.551–0.716	0.003	0.58	60.0%	36.7%
%BF		66.0%	0.578–0.741	<0.001	26.23	64.6%	39.2%
ABSI		59.6%	0.511–0.682	0.031	0.08	61.5%	39.2%
BRI		63.4%	0.551–0.716	0.003	4.92	60.0%	36.7%
CUN-BAE		64.8%	0.565–0.731	0.001	28.31	60.0%	37.5%
CI		63.4%	0.550–0.719	0.003	1.70	60.0%	43.3%
BMI (kg/m^2^)	WC (cm)	91.8%	0.876–0.961	<0.001	25.52	91.8%	25.3%
WHtR		96.2%	0.933–0.991	<0.001	0.52	99.1%	29.3%
%BF		92.9%	0.887–0.970	<0.001	23.84	92.7%	17.3%
ABSI		78.3%	0.714–0.852	<0.001	0.08	77.3%	29.3%
BRI		96.2%	0.933–0.991	<0.001	4.14	94.5%	12.0%
CUN-BAE		92.8%	0.887–0.970	<0.001	25.12	92.7%	16.0%
CI		93.5%	0.899–0.971	<0.001	1.66	90.9%	14.7%

*BMI, Body Mass index; WC, Waist circumference; WHtR, waist-to-height Ratio; %B, percentage body fat; ABSI, a body shape index; BRI, body roundness index; CUN-BAE, Clínica Universidad de Navarra-Body Adiposity Estimator; CI, Conicity index; MetS, metabolic syndrome; HDL-C, high-density lipoprotein cholesterol; FBG, fasting blood glucose; SBP, systolic blood pressure; DBP, diastolic blood pressure; WC, waist circumference*.

Of note is that, based on the CIs that overlapped, there were no significant differences between AUC outcomes for %BF, CUN-BAE, BMI, WHtR, BRI and CI. Moreover, the CIs for the ABSI overlapped with those of the CI, but did not overlap with the rest of the other indices. This showed that, while there was no significant difference between the AUC outcomes for ABSI and CI, there were significant difference between the AUC outcomes for ABSI and those of the other anthropometrical indices. This showed that the ABSI predicted MetS to a significantly lesser degree than the BMI, WHtR, %BF, BRI, and CUN-BAE.

It is further shown that some of these anthropometric indices could not predict the individual risk factors for MetS (predict TG, HDL-c, TG, FBG and BP) since none of these risk factors produced AUCs above 70% in this group of participants, satisfactorily. The only AUCs ≥70% observed was with BMI's and CUN-BAE‘s ability to predict low HDL-c with the cut-off points at 27.74 kg/m^2^ and 26.85, respectively. The rest of the indices only produced AUC outcomes (>60%) with ABSI still performing more poorly than the other indices (AUC <60%). It is further imperative to note the outstanding predictive powers of BMI, %BF, CUN-BAE and CI to predict WC as an important risk factor for MetS with the respective, cut-off points at 25.52 kg/m^2^, 23.84, 25.12 and 1.66. The highest AUC outcomes for screening WC were for the CI and %BF, followed by CUN-BAE then BMI (93.5 and 92.9%, followed by 92.8% then 91.8%), respectively.

According to Table 5, only the DBP and WC could outstandingly predict MetS, with cut-off points of 85.5 mmHg and 99 cm and Sensitivity levels of 70.1 and 81.8%, respectively. The rest of the risk factors managed to predict MetS excellently.

**Table 5:** Optimal cut-off point for components of MetS.

**Variable**	**The area under the curve ROC**	**(95% CI)**	***P*** **-value**	**Optimal cut-off point**	**Sensitivity**	**Specificity**
Triglycerides (mmol/L)	76.7%	0.697–0.837	<0.000	1.11	70.1%	29.6%
HDL-c (mmol/L)	71.2%	0.635–0.789	<0.000	1.03	70.4%	32.5%
Fasting blood glucose (mmol/L)	77.0%	0.703–0.838	<0.000	5.35	79.2%	37.0%
SBP (mmHg)	72.5%	0.650–0.800	<0.000	130.50	70.1%	44.4%
DBP (mmHg)	80.5%	0.739–0.870	<0.000	85.5	70.1%	23.1%
Waist circumference (cm)	83.6%	0.780–0.837	<0.000	99.00	81.8%	29.6%

*SBP, systolic blood pressure; DBP, diastolic blood pressure; HDL-C, high density lipo-protein- cholesterol*.

The outcomes of the logistic regression analyses are shown in Table 6. The unadjusted odds ratios (OR) and adjusted odd ratio (AOR) with 95% confidence intervals (95% CIs) are also presented. While the BMI, WHtR, %BF, BRI and CUN-BAE yielded OR and AOR outcomes (for all the 3 models) that showed significant probability for MetS risk, the OR outcomes for ABSI and CI were not significant. The highest positive (increased) likelihood for MetS risk was with the BRI (almost 2 times more likelihood), than the BMI (almost 1.3 times more likelihood), followed by %BF and CUN-BAE (1.214 and 1.215 more likelihood, respectively). All the *p* < 0.001 and the positive likelihoods remained after removing the confounding effects of age, race, marital status, driving experience in years, education, smoking, alcohol intake and physical activity. The WHtR on the other hand yielded a negative (0.977 less likelihood) for MetS risk (where the *p*-value for OR was < 0.001). This less likelihood persisted after removing the confounding effects of age, race, marital status, driving experience in years, education, smoking, alcohol intake and physical activity.

**Table 6:** The risk for metabolic syndrome among South African males aged 20 years and older by anthropometric indices.

	**Unadjusted**			**Adjusted OR model 1**			**Adjusted OR model 2**			**Adjusted OR model 3**		
**Anthropometric indices**	**Crude OR**	**95% CI**	***p*** **-Value**	**Crude** **OR**	**95% CI**	***p-*** **Value**	**Crude OR**	**95% CI**	***p*** **-Value**	**Crude** **OR**	**95% CI**	***p*** **-Value**
BMI (kg/m^2^)	1.277	1.182–1.379	<0.001	1.261	1.166–1.363	<0.001	1.271	1.170–1.382	<0.001	1.269	1.165–1.382	<0.001
WHtR (cat)	0.023	0.003–0.174	<0.001	0.026	0.003–0.196	<0.001	0.028	0.004–0.215	0.001	0.030	0.004–0.232	0.001
%BF	1.214	1.145–1.288	<0.001	1.213	1.137–1.294	<0.001	1.221	1.140–1.308	<0.001	1.220	1.136–1.309	<0.001
ABSI (cat)	2.853	0.254–32.041	0.395	1.663	0.145–19.041	0.683	2.228	0.170–29.270	0.540	1.754	0.123–25.036	0.679
BRI	1.922	1.549–2.386	<0.001	1.817	1.466–2.250	<0.001	1.860	1.478–2.342	<0.001	1.819	1.442–2.294	<0.001
CUN-BAE	1.215	1.146–1.288	<0.001	1.202	1.132–1.276	<0.001	1.210	1.136–1.289	<0.001	1.210	1.134–1.292	<0.001
CI cat	2.853	0.254–32.041	0.395	1.663	0.145–19.041	0.683	2.228	0.170–29.270	0.540	1.754	0.123–25.036	0.679
**MetS risk factors**												
Triglycerides (mmol/L)	5.468	2.879–10.387	<0.001	5.883	2.957–11.703	<0.001	6.205	2.986–12.892	<0.001	7.370	3.337–16.279	<0.001
HDL-c (mmol/L)	0.089	0.026–0.308	<0.001	0.085	0.023–0.320	<0.001	0.079	0.020–0.308	<0.001	0.067	0.016–0.288	<0.001
FBG (mmol/L)	1.869	1.402–2.492	<0.001	1.765	1.317–2.366	<0.001	1.693	1.254–2.286	0.001	1.770	1.295–2.419	0.001
SBP (mmHg)	1.063	1.037–1.089	<0.001	1.064	1.037–1.092	<0.001	1.070	1.041–1.101	<0.001	1.067	1.037–1.098	<0.001
DBP (mmHg)	1.121	1.080–1.163	<0.001	1.117	1.075–1.161	<0.001	1.121	1.077–1.167	<0.001	1.119	1.073–1.168	<0.001
Hypertension (cat)	0.099	0.049–0.201	<0.001	0.108	0.052–0.223	<0.001	0.092	0.042–0.203	<0.001	−0.102	0.046–0.226	<0.001
WC (cm)	1.097	1.065–1.129	<0.001	1.090	1.059–1.122	<0.001	1.092	1.059–1.126	<0.001	1.090	1.056–1.124	<0.001

*Three logistic regression models were applied: model 1, adjusted for age; model 2, adjusted for age, race, marital status, driving experience in years, and education; and model 3, further adjusted for age, race, marital status, driving experience in years, education, smoking, alcohol and physical activity. BMI, Body Mass index; WC, Waist circumference; WHtR, waist-to-height Ratio; %BF, percentage body fat; ABSI, a body shape index; BRI, body roundness index; CUN-BAE, Clínica Universidad de Navarra-Body Adiposity Estimator; CI, Conicity index; MetS, metabolic syndrome; HDL-C, high-density lipoprotein cholesterol; FBG, fasting blood glucose; SBP, systolic blood pressure; DBP, diastolic blood pressure; WC, waist circumference. Cat: categorical variable, Hypertension: systolic blood pressure ≥ 130 mmHg and/or diastolic blood pressure ≥ 85 mmHg, WHtR: (> 0.5), CI: (>1.25), ABSI: > 0.086*.

Moreover, the TG, FBG, SBP, DBP and WC yielded positive outcomes (increased likelihood of 5.5, 1.9, 1.1, 1.2 and 1.1 times) for MetS risk, respectively. All the *p* <0.01 and these remained after adjusting for age, race, marital status, driving experience in years, education, smoking, alcohol intake and physical activity. The HDL-c and hypertension on the other hand yielded negative outcomes (reduced likelihood of 0.911 and 0.901) for MetS risk, respectively. All the *p* < 0.001 and these remained after adjusting for age, race, marital status, driving experience in years, education, smoking, alcohol intake and physical activity.

We further investigated how the combinations of two indices behaved in predicting MetS among study participants in Table 7. It was shown that all combination of two indices had significantly better performances in predicting MetS. e.g., One unit increase in the combination of BRI and WHtR increased two times chances of MetS (OR: 1.871 95% CI 1.522–2.302, *p* < 0.001) for unadjusted. While in the adjusted model 1, increased 1.7 times chances of MetS incident (OR 1.773 95CI 1.444–2.177, *p* < 0.001).

**Table 7:** The unadjusted and adjusted odds ratios (ORs) of the combination BMI and BRI, BMI and WHtR, and BRI and WHtR for prediction of MetS and its risk factors.

**Unadjusted**									
	**BMI and BRI**			**BMI and WHtR**			**BRI and WHtR**		
	**OR**	**95% CI**	***P*** **-value**	**OR**	**95% CI**	***P*** **-value**	**OR**	**95% CI**	***P*** **-value**
MetS	1.211	1.141–1.286	<0.001	1.274	1.180–1.375	<0.001	1.871	1.522–2.302	<0.001
Triglycerides	1.072	1.029–1.117	<0.001	1.095	1.038–1.156	<0.001	1.222	1.069–1.397	0.003
HDL-c	1.088	1.045–1.133	<0.001	1.121	1.062–1.182	<0.001	1.256	1.097–1.439	<0.001
FBG	1.051	1.015–1.087	0.005	1.065	1.019–1.114	0.005	1.173	1.041–1.321	0.009
Hypertension	1.056	1.021–1.093	0.002	1.073	1.026–1.123	0.002	1.184	1.053–1.332	0.005
WC	1.564	1.388–1.761	<0.001	1.601	1.419–1.806	<0.001	9.955	5.234–19.010	<0.001
**Adjusted OR model 1**									
MetS	1.197	1.128–1.277	<0.001	1.258	1.165–1.358	<0.001	1.773	1.444–2.177	<0.001
Triglycerides	1.072	1.028–1.118	0.001	1.094	1.036–1.156	0.001	1.219	1.063–1.397	0.005
HDL-c	1.092	1.047–1.138	<0.001	1.125	1.064–1.187	<0.001	1.267	1.101–1.458	<0.001
FBG	1.039	1.004–1.076	0.030	1.051	1.005–1.100	0.030	1.124	0.995–1.269	0.060
Hypertension	1.048	1.012–1.086	0.009	1.063	1.016–1.113	0.008	1.150	1.020–1.297	0.023
WC	1.584	1.396–1.797	<0.001	1.629	1.429–1.857	<0.001	9.783	5.091–18.800	<0.001
**Adjusted OR model 2**									
MetS	1.203	1.130–1.280	<0.001	1.264	1.167–1.370	<0.001	1.777	1.436–2.199	<0.001
Triglycerides	1.072	1.027–1.120	0.002	1.094	1.034–1.158	0.002	1.225	1.065–1.408	0.004
HDL-c	1.094	1.047–1.142	<0.001	1.127	1.065–1.193	<0.001	1.266	1.097–1.460	0.001
FBG	1.039	1.003–1.076	0.035	1.050	1.004–1.100	0.035	1.122	0.993–1.267	0.065
Hypertension	1.049	1.011–1.088	0.010	1.064	1.015–1.115	0.010	1.151	1.018–1.301	0.025
WC	1.581	1.394–1.795	<0.001	1.625	1.425–1.853	<0.001	9.979	5.158–19.307	<0.001
**Adjusted OR model 3**									
MetS	1.191	1.118–1.269	<0.001	1.250	1.153–1.356	<0.001	1.710	1.384–2.113	<0.001
Triglycerides	1.072	1.026–1.121	0.002	1.094	1.033–1.160	0.002	1.274	1.059–1.415	0.006
HDL-c	1.097	1.047–1.148	<0.001	1.131	1.066–1.201	<0.001	1.260	1.088–1.460	0.002
FBG	1.036	0.999–1.074	0.054	1.047	0.999–1.097	0.055	1.114	0.983–1.262	0.092
Hypertension	1.040	1.002–1.080	0.039	1.053	1.003–1.106	0.036	1.115	0.981–1.266	0.096
WC	1.610	1.404–1.846	<0.001	1.639	1.427–1.883	<0.001	10.798	5.362–21.744	<0.001

*Three logistic regression models were applied: model 1, adjusted for age; model 2, adjusted for age, race, marital status, driving experience in years, and education; and model 3, further adjusted for age, race, marital status, driving experience in years, education, smoking, alcohol and physical activity. BMI, Body Mass index; WC, Waist circumference; WHtR, waist-to-height Ratio; BRI, body roundness index; MetS, metabolic syndrome. HDL-C, high-density lipoprotein cholesterol; FBG, fasting blood glucose; WC, waist circumference*.

**Figure 3:**
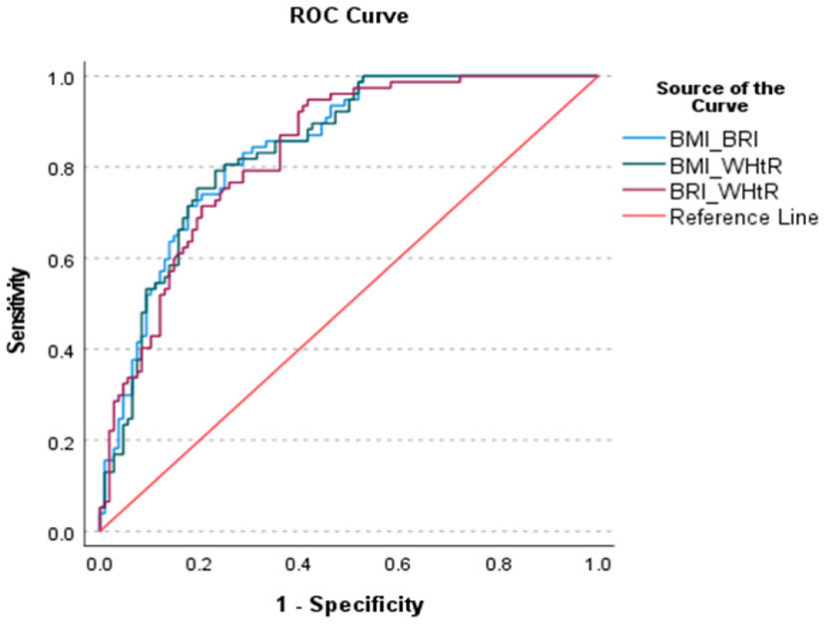
ROC curve of the various combination of two anthropometric indices fir identifying the MetS.

Since we had the evidence that the anthropometric indices would predict the risk of MetS, we now investigated how much it could be improved with combinations of indices using AUC. Figure 3 and Table 8 show the AUC's of various combinations of two indices for predicting MetS. It was obvious that the predictive capacity for MetS with two indices was much better than that with a single index. For example, the AUC of BMI and BRI, BMI and WHtR and BRI and WHtR for predicting MetS were 0.843, 0.839 and 0.832, respectively.

**Table 8:** Area under the curves (AUC) for the various combinations of two anthropometric indices for identifying MetS.

	**The area under the curve ROC**	***P*** **-value**	**95% CI**	
BMI and BRI	0.843	<0.001	0.788	0.898
BMI and WHtR	0.839	<0.001	0.783	0.895
BRI and WHtR	0.832	<0.001	0.775	0.889

*BMI, Body Mass index; WC, Waist circumference; WHtR, waist-to-height Ratio; BRI, body roundness index; MetS, metabolic syndrome. HDL-C, high-density lipoprotein cholesterol; FBG, fasting blood glucose; WC, waist circumference*.

## Discussion

The current study aimed to examine the power of anthropometric indices such as the BMI, WC, WHtR, ABSI, BRI, %BF, CI, and CUN-BAE to predict MetS, and determine the cut-off points to identify male South African taxi drivers at risk of MetS. The mean age of the participants was 39.84 years. Overall, more than 41% of the participants had MetS. Participants presenting with MetS were significantly older than those without MetS. The highest AUC outcomes for screening MetS were for the %BF and CUN-BAE, followed by the WC, WHtR and BMI, and lastly by the BRI. All these anthropometric indices had outstanding discriminatory powers for predicting MetS since their AUC outcomes were above 80%. While all the indices had outstanding capabilities to predict MetS, ABSI was considered a poor indicator of MetS when compared to the rest of the indices. In terms of the abilities of the indices to predict the risk of elevated TGs, FBG and BP, as well as reduced HDL-c, only the BMI and CUN-BAE produced AUC outcomes that were above 70%. Finally, based on the logistic regression models shown in the current paper, the taxi drivers that presented with abnormal levels of BMI, WHtR, %BF, BRI, CUN-BAE, TG, HDL-c, FBG, SBP, DBP and WC displayed an increased risk of MetS.

The prevalence for MetS in our study appears to be high (41.6%) when compared to other documented South African studies. In fact, Motala et al. (21); Motala et al. (22) and Sekgala et al. (24) found MetS to be 7.9, 10.5 and 8.6% in rural South African men, respectively. Peer et al. (23), on the other hand, reported a 17.9% prevalence of MetS in black men living in urban townships in Cape Town, South Africa. Our results are also higher than the prevalence of 17.1% observed by Mabetwa et al. (52) among taxi drivers operating in the City of Tshwane and the prevalence of other international studies among occupational drivers for example Chen et al. (2) (6.23%), Montazerifar et al. (3) (20.0%) and Saberi et al. (4) (35.9%). However, we need to mention that the prevalence of MetS might be different according to the definition used to determine MetS. Several international studies define MetS using the Adult Treatment Panel III for Asians which considers any three of MetS clusters while for Sub-Saharan Africa (SSA) the IDF European definition is used which considers WC and any two clusters of cardiometabolic disorders.

The increased prevalence might also be attributed to the fact that almost 60% of the taxi drivers participating in the current research presented with central obesity while the majority the taxi drivers with central obesity also presented with MetS. Though comparable to the 50% of international male long distance and long duration drivers observed by Hirata, et al. (29) and Saberi et al. (4), the current abnormal WC prevalence outcome is still the highest when compared to all other outcomes we could review from literature. In the current study it has also been shown that WC correlates well with other anthropometric indices including the BMI, WHtR, %BF, BRI, CUN-BAE (AUC >90%).

Because central obesity explains fat mass that lines internal organs, if in excess, it is likely to disturb the natural functioning of these organs, hence it is detrimental to human health. According to available South African (21–24, 52, 53) and international (4, 54) studies, central obesity is more prevalent in middle age to older men, and it positively correlates with other body composition outcomes including abnormal BMI, WC, waist to hip ratio (WHpR), %BF and all sorts of CVD risk factors and MetS.

Other notable outcomes of the current study indicated that indices which determine body fat distribution, %BF, CUN-BAE, WHtR and BRI, specifically showed outstanding discriminatory power for predicting the risk of MetS. These findings are corroborated by other cross-sectional South African (53) and international (34, 55) studies conducted among different ethnic groups of men operating in the driving industry. Moreover, in line with our current findings, Głuszek et al. (34) showed that the ABSI index showed the lowest discriminatory powers to predict MetS when compared to other anthropometrical indices with an AUC of 60%. Zhang et al. (56), also showed the weakness of CI in predicting MetS in Chinese male adults with the AUC of 66%. These outcomes can be attributed to the fact that the algorithms for ABSI and CI consider BMI and body weight, respectively. Evidence suggests that the BMI and body weight do not consider the distribution of adipose tissue. Earlier presented evidence indicate that MetS is sensitive to central obesity (57, 58). Moreover, Głuszek et al. (34), Mongraw-Chaffin et al. (59), and Heymsfield et al. (60) have eloquently argued that the cut-off points for the BMI and weight do not consider the individual's ethnicity, gender and age-group, hence they appear to be less sensitive in predicting MetS, especially in a group of participants in the current study, who were males of whom the majority were of black decent.

There is growing evidence (53, 61) that highly recommend WC and WHtR as the best anthropometric indices to be used in the diagnosis of MetS and its risk factors. Both these indices have been shown to produce AUC outcomes that are >80% when detecting MetS and its risk factors including diabetes mellitus. Moreover, Rajput et al. (62) previously argued that the WHtR can be used independently as a universal screening tool to identify individuals at high risk of developing metabolic complications, regardless of the individuals' gender or geographical location. Other researchers have also advocated the importance of using the WC, WHtR, BRI and CUN-BAE in the diagnosis of cardiometabolic disorders and MetS (36, 45, 63, 64). According to Thomas et al. (36), the BRI was created to measure body fat and the percentage of visceral adipose tissue by using WC and height in the algorithm. Pairing WC and height in the same algorithm elevates the discriminatory power of the index to predict the risk of MetS. It should also be noted that, according to Maessen et al. (65), the BRI has a relatively strong correlation (r = 0.999) with WHtR among the Dutch population. Several other studies have confirmed the BRI's ability to identify the risk of MetS in both men and women (56, 66, 67).

Prospective studies (68–70) have highlighted the usefulness of anthropometric indices to identify individuals at risk of cardiometabolic disorders such as hypertension, elevated blood glucose and blood lipids. However, none of the anthropometrical indices produced AUCs above 70% in the calculations undertaken to predict FBG, TGs, hypertension, DBP, and SBP, with the exceptions being the BMI's and CUN-BAE's ability to predict low HDL-c (where both AUC outcomes were 70%), with the cut-off points at 27.74 kg/m^2^ and 26.85, respectively.

Similar results were reported by Głuszek et al. (34) where CUN-BAE, BMI, and WC in men (AUC = 0.734, 0.728, and 0.728, respectively) had the highest discriminatory power for the identification of at least one MetS component. Contrary to our outcomes, none of the anthropometric indices were shown to predict the incidence of low HDL-c in the study by Latifi et al. (71). It is unclear why such contrasting outcomes were observed. However, it needs to be acknowledged that these studies were undertaken, to a large extent, in different ethnic groups, genders, age groups and geographic location.

The current research outcomes also established new anthropometric indices' cut-off points to predict MetS among South African taxi drivers. For instance, the cut-off point established for BMI (28.25 kg/m^2^) in the current study seems lower than 30 kg/m^2^ recommended by the IDF (49). Al-Odat et al. (72) found lower cut-off points of 28.4 kg/m^2^ in their research conducted in the male Jordan population while Ofer et al. (73) reported cut-off points of 27 kg/m^2^ in the retrospective, observational, cohort-based study. Even though several papers, including the current manuscript highlight the limitations of using BMI independently (28, 74, 75) to predict MetS, BMI can still be a very user friendly, non-invasive and affordable tool to measure adiposity and predict other of chronic metabolic diseases.

In terms of WC cut-off points to predict MetS, ours were within the range recommended by the IDF and WHO. In fact, 99 vs. 94 cm and 102 cm, respectively, were observed in the current study. Moreover, the cut-off point of 0.55 for WHtR reported falls within the range of 0.51 to 0.58 as recommended by the IDF and Głuszek et al. (34). Moreover, several studies (56, 76–78) recommend a WHtR cut-off value of >0.5 as a simple and reliable outcome to identifying those individuals (male and female) who are at an increased risk of metabolic complications.

According to the IDF (2005), the European cut-off point for abdominal obesity should be 94 cm for men (49), whereas the WHO cut-off point is 102 cm for men (47). These figures have been found to be highly correlated with a BMI of around 30 kg/m^2^.

In the current study, we also observed that the %BF and the CUN-BAE were better predictors of MetS (79), compared to BMI, WHtR, CI and BRI. We could attribute these outcomes to the fact that the total body fat predicts metabolic disorders more precisely than other anthropometric indices derived from WC (80). In fact, according to Lear et al. (81) % BF highly correlates with visceral adipose tissue (VAT) hence the excess body fat is primarily responsible for the health consequences associated with obesity (55, 82, 83).

Similar to Macek et al. (84) findings (25.8%), the optimal cut-off point for %BF in the current study was 25.29%. These outcomes were expected given that in the afore-mentioned two studies, men of a similar age group were studied. Similarly, Joseph et al. (85) indicated that 25.5 %BF was sufficient to predict cardiovascular risk in Asian Indian men. Our cut-off point was also similar to the cut-off point recommended by the WHO (25%). However, 25.29% is lower than the outcomes observed in the improving interMediAte RisK management (MARK) study (cut-off point of 31.22%) by Gomez-Marcos et al. (55). The differences could probably be ascribed to the different age groups studied. Gomez-Marcos et al. (55) studied 35–74-year old participants, while in the current research taxi drivers 20 years and older were included.

Finally, based on the logistic regression models shown in the current paper, abnormal BMI, WHtR, %BF, BRI, CUN-BAE, TG, FBG, SBP, DBP and WC outcomes showed increased likelihood for MetS while abnormal HDL-c outcomes showed less likelihood for MetS. There is South African (52, 53) evidence on men and taxi drivers including long distance and long duration drivers, respectively to corroborate these outcomes. However, the outcome in the current study that suggested that hypertensive taxi drivers had decreased likelihood of MetS was surprising. Nonetheless, blood pressure results further showed that elevated DBP and SBP were significantly positively associated with the likelihood of developing MetS among participants. This outcome seems similar to the study of Mabetwa et al. (52). Even though not significant (*p* = 0.117), taxi drivers with hypertension in Mabetwa et al. (52) study were 45% less likely to present with MetS (CI: 0.261–1.161). The take-home messages from the current study are summarized in Box 1.

Box 1 **Take-home messages from the current research.**

- Based on the current study, Overall, 41.6% of the South African men taxi drivers had MetS.
- The mean values for BMI, WC, WHtR, %BF, BRI, CUN-BAE, ABSI and CI were significantly higher in older participants and those that presented with MetS compared to younger participants without MetS.
- Participants who presented with MetS had higher mean values for triglycerides (1.88 vs 0.96), FBG (7.87 vs. 5.33), SBP (141.47 vs. 127.40) and WC (110.83 vs. 90.72) as compared to those without MetS.
- Overall, 20.8, 51.6, 51.7, 59.7, 43.3, 35.5 and 59.9% of the participants had abnormal Triglyceride, HDL-c, FBG, SBP, DBP, Hypertension and WC, respectively.
- The highest AUC outcomes for screening MetS were for the %BF and CUN-BAE and then followed by the WC, BMI and WHtR, and lastly the BRI (84.8 and 84.6%, and then followed by 83.8 and 83.2%, and lastly the 83.2%, respectively).

➢ This means that all these anthropometrical indices had outstanding discriminatory power for predicting MetS since their AUC and sensitivity levels were above 80%.

- The BMI, WHtR, %BF, BRI, and CUN-BAE, had cut-off points for detection of MetS in South African men at 28.25 kg/m^2^, 0.55, 25.29%, 4.55, and 27.10, respectively.
- While the CI only showed the excellent AUC (76.2%) for predicting the MetS with the cut-off point of 1.70 and the sensitivity of 74%.
- Some of these anthropometric indices could not satisfactorily predict the individual risk factors for MetS (i.e., predict TG, HDL-c, TG, FBG and BP).

➢ This means that none produced the AUCs that were above 70% in this group of participants.
➢ The only acceptable outcome (AUCs ≥70%) observed was with BMI's and CUN-BAE‘s ability to predict HDL-c with the cut-off points at 27.74 kg/m^2^ and 26.85, respectively.
➢ There was outstanding predictive powers of BMI, %BF, CUN-BAE and CI to predict WC with the cut-off point at 25.52 kg/m^2^, 23.84, 25.12 and 1.66, respectively.
➢ This means that all these anthropometrical indices had outstanding discriminatory power for predicting WC since their AUCs and sensitivity values were all above 90%.
➢ DBP and WC showed outstanding predictive powers to diagnose MetS with cut-off points of 85.5 mmHg and 99 cm, respectively.

- We observed the highest positive likelihood for BRI and BMI to increase the incidence of MetS in the unadjusted and all the adjusted models.
- Increased in CUN-BAE and %BF were positively associated with likelihood of MetS incidence.
- High triglycerides had a greater risk of increasing MetS in both adjusted and unadjusted models.

### Limitations

While several strengths of the current study are outlined above, there are limitations that should be considered when interpreting the current outcomes. Firstly, this study was the cross-sectional design which cannot infer causality. Secondly, the sample size because of the specific nature of the chosen participants (male and taxi drivers), therefore, as only male taxi drivers that were recruited conveniently are included, the outcomes obtained can only be generalizable in populations with similar characteristics as the current participants. Possible reasons for the high prevalence of MetS in our analysis might be influenced by genetic variation and epigenetic factors (86), adipose-related hormonal and immunological reactions can exacerbate metabolic disorders, such as dyslipidemia and high blood pressure (87). The main environmental factors influencing the expression of genes involved in the occurrence of MetS are eating habits and physical activity (88). Diets high in fat, particularly saturated fat, with a high glycemic index and low fiber content can increase the risk of MetS. Therefore, not all MetS cases can be characterized by high anthropometric indices as MetS can be linked not only to excess adipose tissue but also to its location.

## Conclusion

The results of our study confirmed the usefulness of BMI, WHtR, %BF, BRI, and CUN-BAE for identifying MetS in male drivers, whereas ABSI was found to be the weakest predictor of the syndrome.

Therefore, the cut-off points proposed in this study provide an earlier diagnosis of MetS than the commonly accepted obesity criterion (BMI ≥30 kg/m^2^). In our analysis, we included the MetS definition (three of five components according to the IDF) and anthropometric indices excluding WC. To avoid a late diagnosis of MetS, consideration should be given to setting cut-off points for the indicators in question that would allow people with only one MetS component to be diagnosed. This data might be clinically significant, as anthropometric index reference thresholds can be used to identify those adults who are at high metabolic risk. Additionally, these results highlight the usefulness of BMI, WHtR, %BF, BRI, and CUN-BAE for public health purposes given their higher accuracy and low cost for measurement.

## Data availability statement

The raw data supporting the conclusions of this article will be made available by the authors, without undue reservation.

## Ethics statement

The studies involving human participants were reviewed and approved by Ms. Patricia Josias Research Ethics Committee Officer University of the Western Cape. The patients/participants provided their written informed consent to participate in this study.

## Author contributions

MDS and ZJ-RM: conceptualization and funding acquisition. MDS: formal data analysis, methodology, and writing-original draft. ZJ-RM and MO: supervision and writing-review and editing. BM: biochemical analysis. All authors contributed to the article and approved the submitted version.

## Funding

The work reported herein was made possible through Cochrane South Africa, the South African Medical Research Council (SAMRC) under the Collaboration for Evidence Based Healthcare and Public Health in Africa (CEBHA+) Scholarship Programme. CEBHA+ receives funding from the Federal Ministry for Education and Research (Bundesministerium fBildung und Forschung, BMBF), Germany, through the BMBF funding of Research Networks for Health Innovation in Sub-Saharan Africa (Funding No. 81203621), Non-Communicable Diseases Research Unit (NCD-RU) of the SAMRC, and Human and Social Capabilities (HSC) division of the Human Science Research Council (HSRC).

## Conflict of interest

The authors declare that the research was conducted in the absence of any commercial or financial relationships that could be construed as a potential conflict of interest.

## Publisher's note

All claims expressed in this article are solely those of the authors and do not necessarily represent those of their affiliated organizations, or those of the publisher, the editors and the reviewers. Any product that may be evaluated in this article, or claim that may be made by its manufacturer, is not guaranteed or endorsed by the publisher.

## Author disclaimer

The content hereof is the sole responsibility of the authors and does not necessarily represent the official views of SAMRC or the funders.
