# Supplementary figures and images for: Anthropometric indices and cut-off points for screening of metabolic syndrome among South African taxi drivers
Source: Front Nutr. 2022 Aug 11;9:974749. doi: 10.3389/fnut.2022.974749 (PMC9406286; doi:10.3389/fnut.2022.974749)

**Appendix**


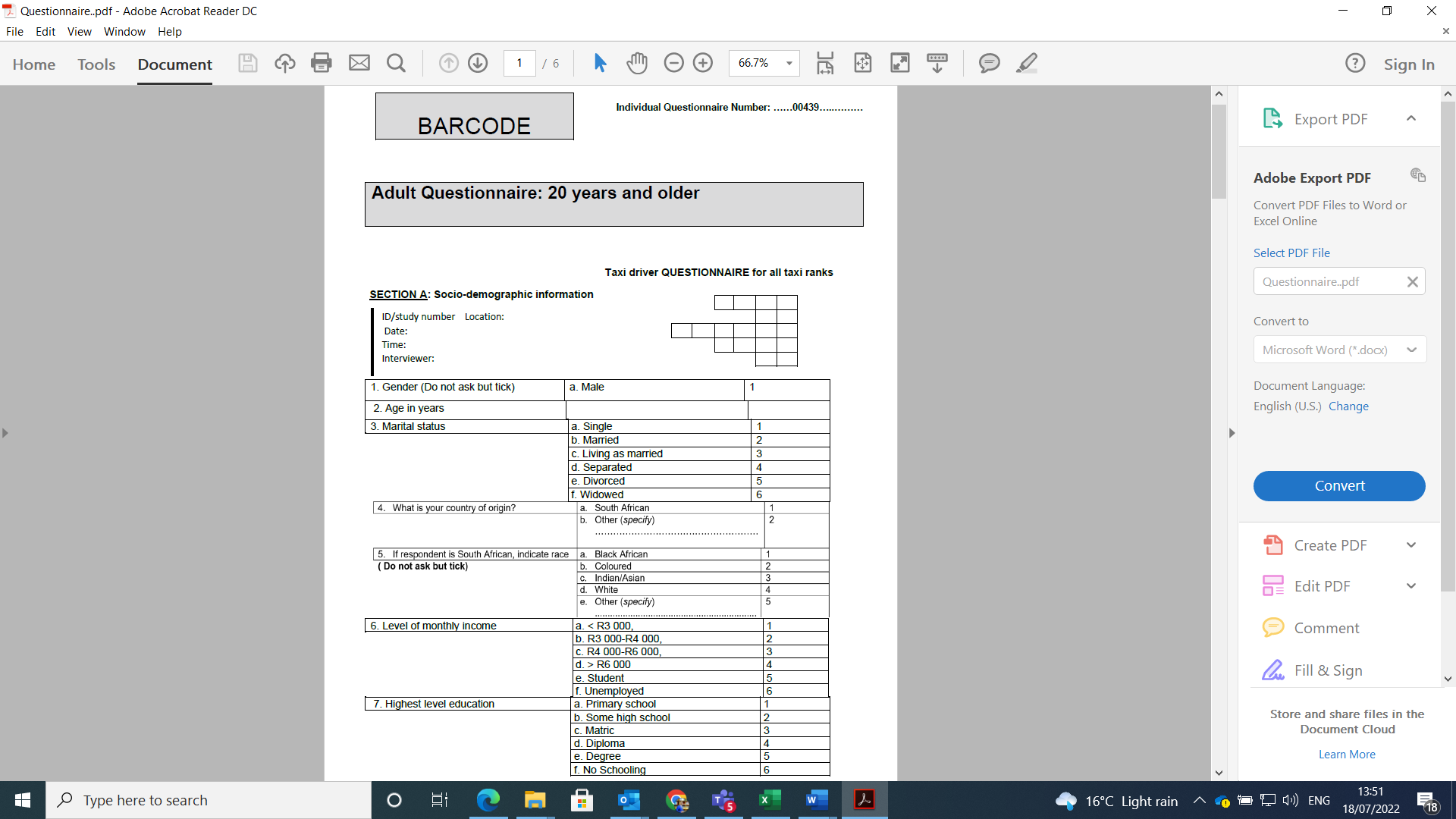


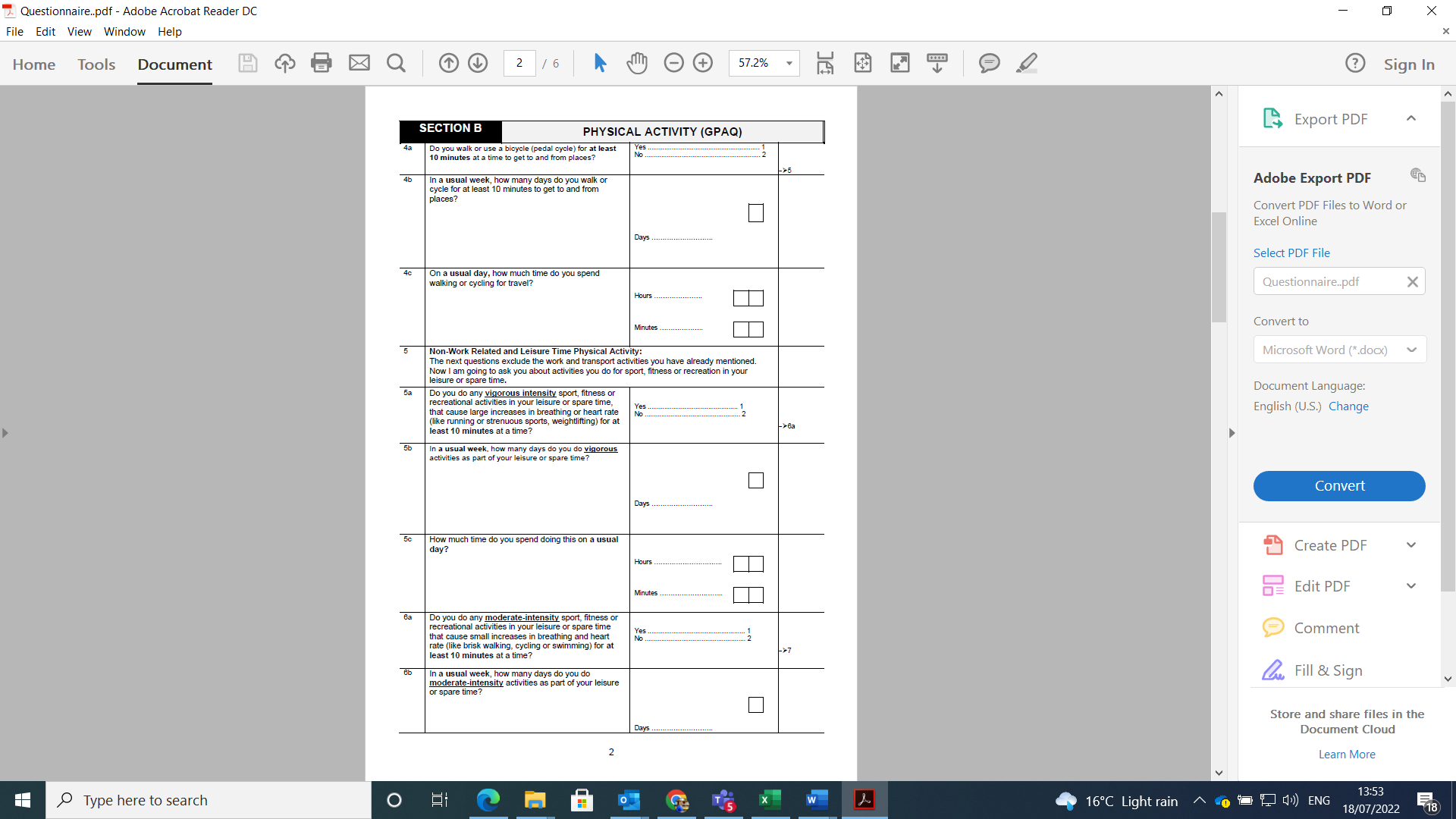

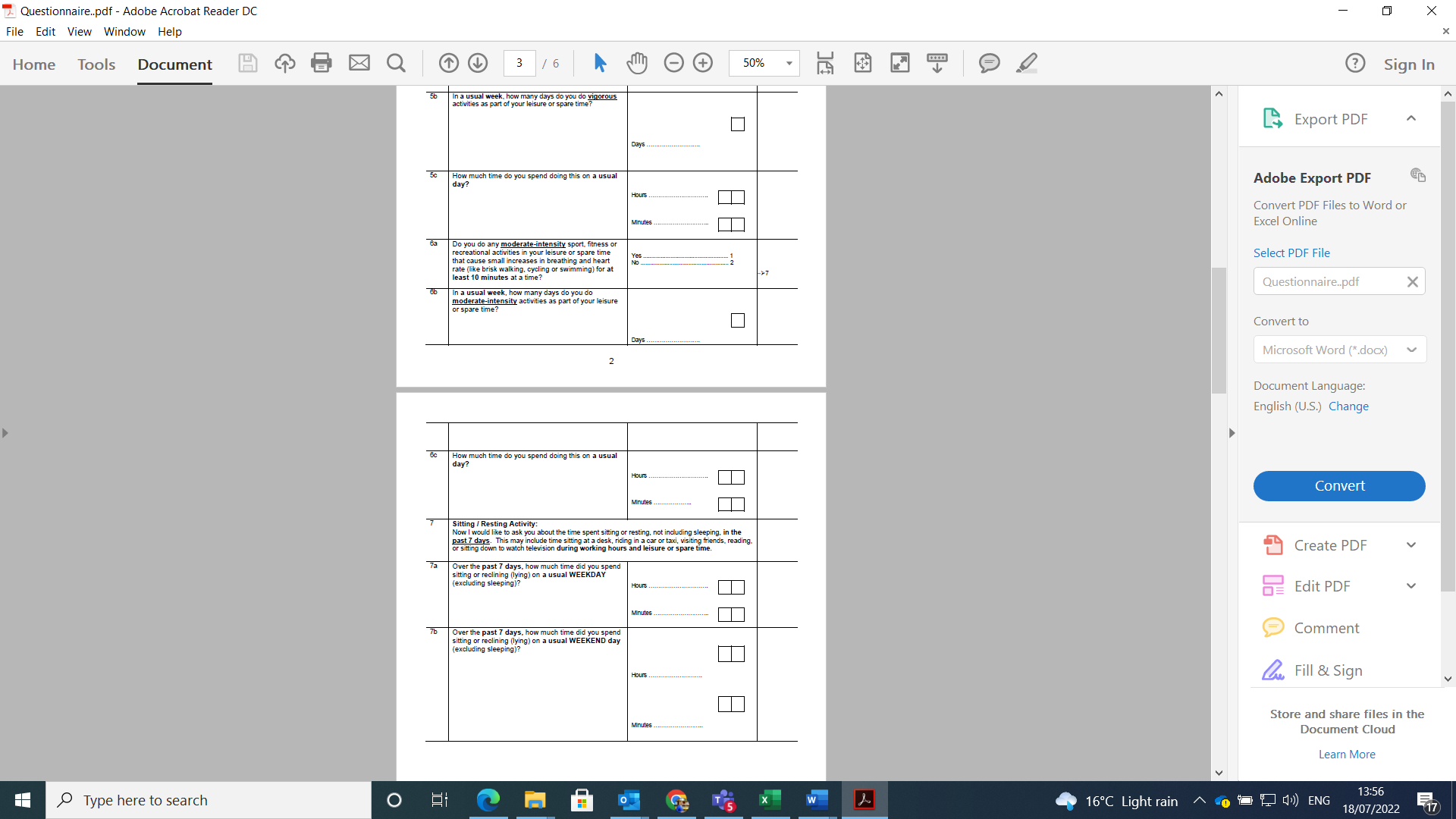


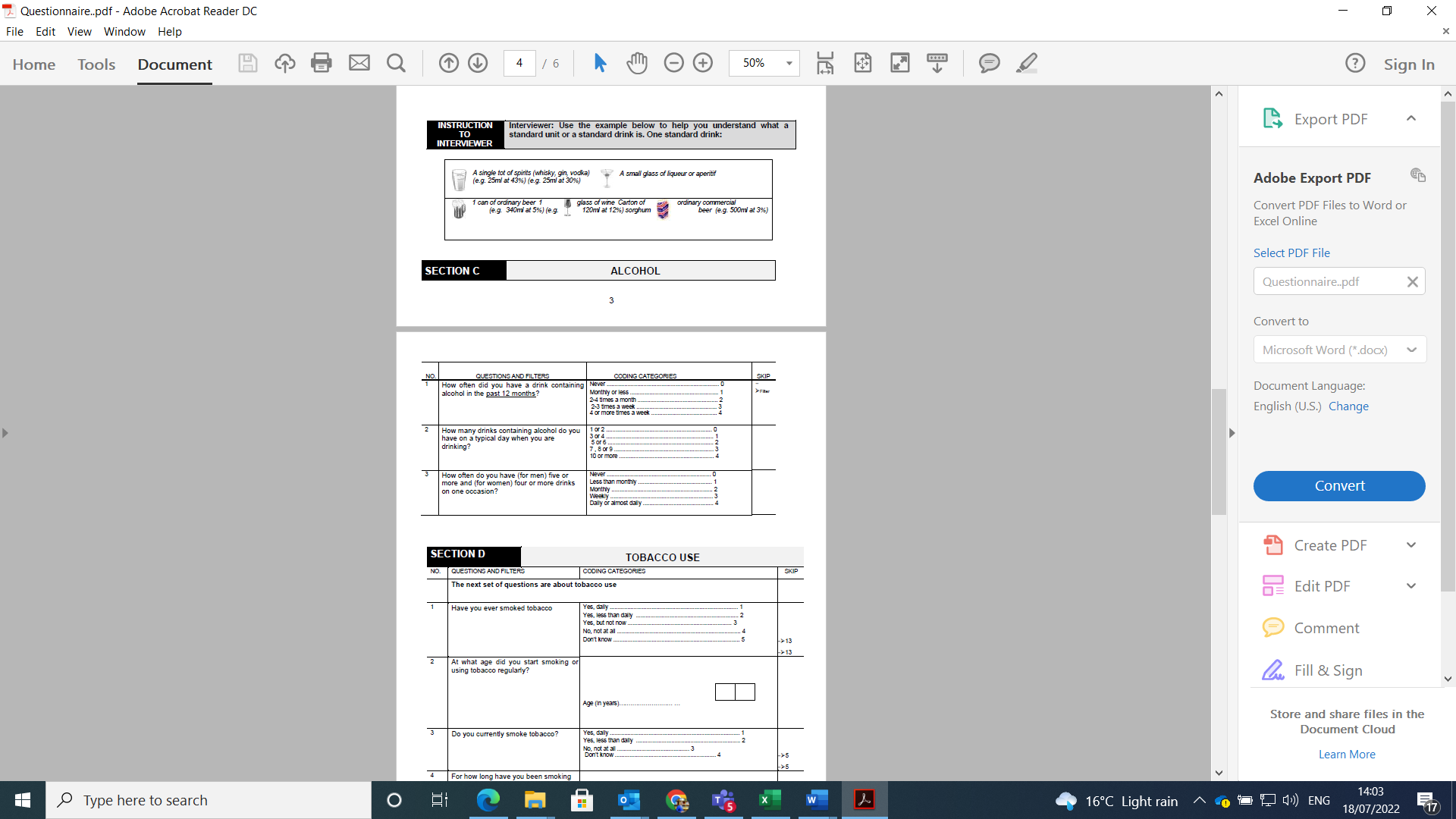


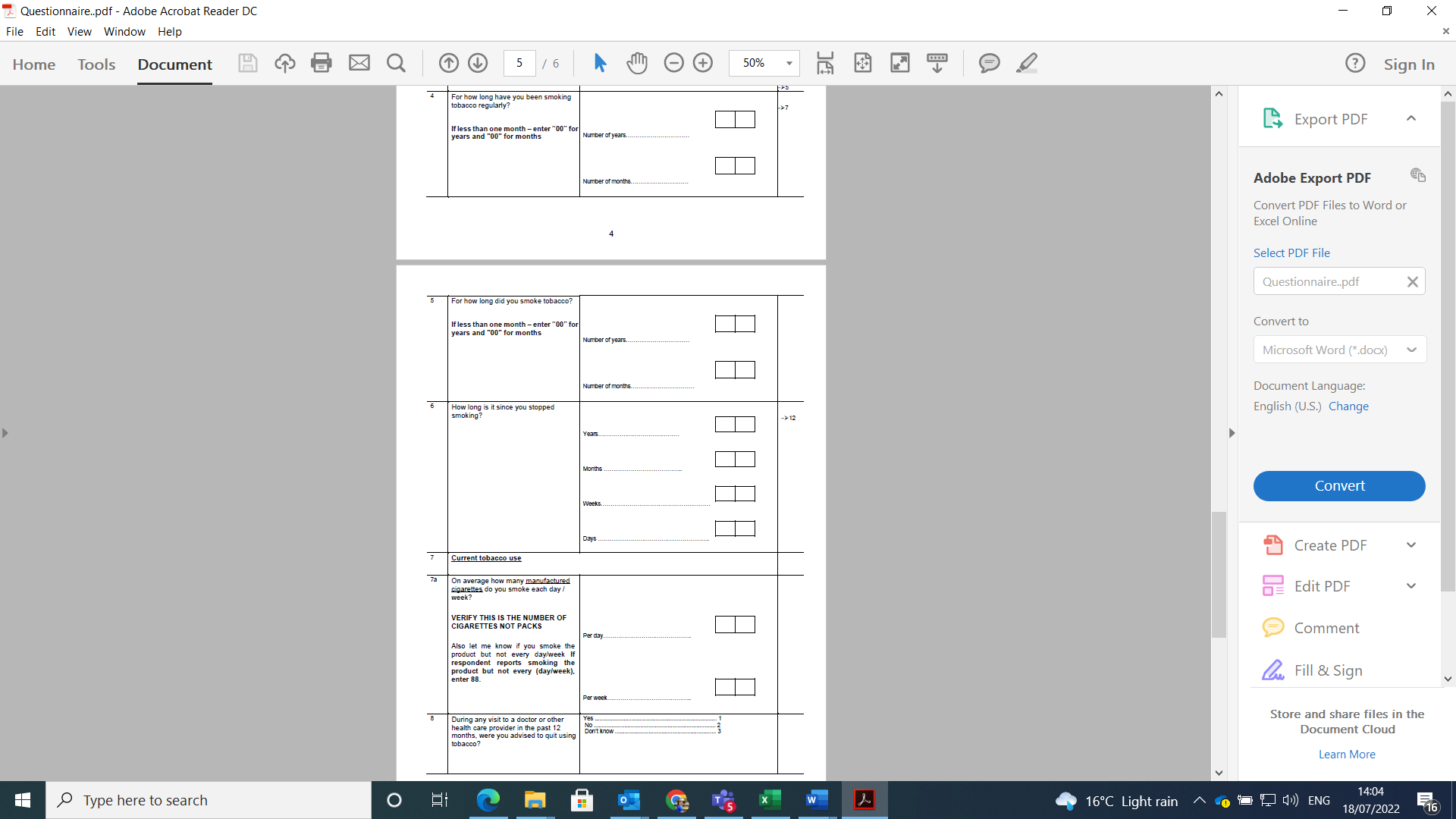


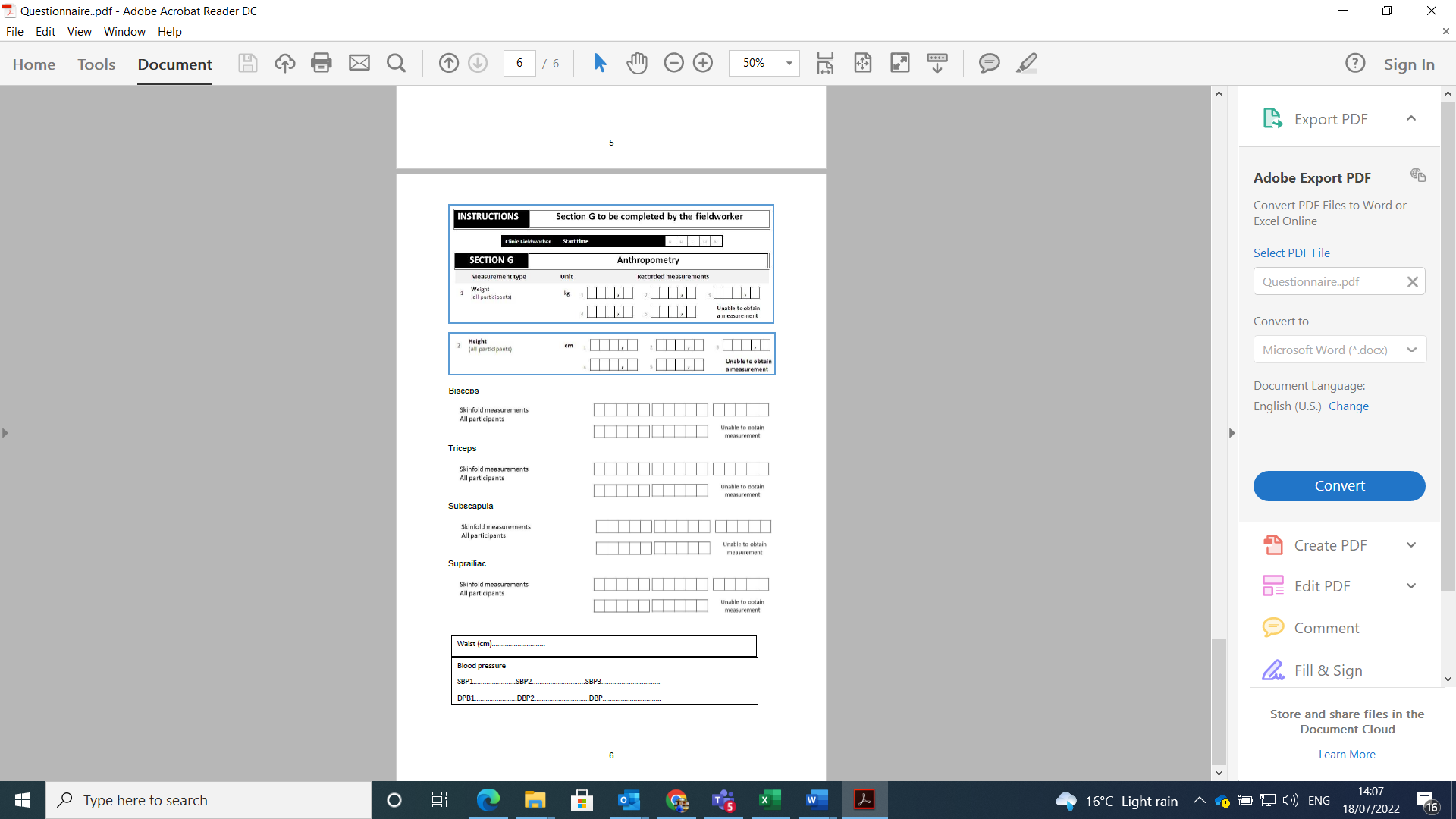

Supplement: Supplementary file 1 [file Data_Sheet_1.docx]
